# Different words for stroke: the same concept? an analysis of associated symptoms and intended reaction in Brazil

**DOI:** 10.1186/s12883-023-03327-y

**Published:** 2023-07-18

**Authors:** Mário Luciano de Mélo Silva Júnior, Ana Gabriella Camelo Oliveira, Weslley Medeiros Gois, Matheus Franco Andrade Oliveira, Lourdes Maria Dantas de Góis, Lucas Pereira Ferreira, Marcos Vinícius de Souza Vilanova

**Affiliations:** 1 Medical Sciences Center, Division of Neuropsychiatry, Universidade Federal de Pernambuco, Recife, Pernambuco, Brasil; 2 Neurology Unit, Hospital da Restauração, Recife, Pernambuco, Brasil; 3 Medical School, Uninassau, Recife, Pernambuco, Brasil; 4 Universidade Federal de Sergipe, curso de graduação em Medicina, Lagarto, Sergipe, Brasil; 5 Escola Bahiana de Medicina e Saúde Pública, curso de graduação em Medicina, Salvador, Bahia Brasil; 6 Universidade Potiguar, curso de graduação em Medicina, Natal, Rio Grande do Norte Brasil

**Keywords:** Cerebrovascular disorders, Stroke, Signs and symptoms, Health knowledge, attitudes, practice, Surveys and questionnaires

## Abstract

**Background:**

Different names for stroke might mislead physicians and emergency medical service workers. This study aimed to assess the different words for stroke in Brazil and both intended response and related symptoms associated with those names.

**Methods:**

Cross-sectional study enrolling healthy individuals from urban areas in Northeast of Brazil for an open-ended survey. We presented a typical clinical case of a stroke (an elderly who had sudden onset of hemiparalysis and slurred speech) and asked “what is happening?”, “what would you do?” and “which other symptoms could happen in this condition?”.

**Resuts:**

From 1,475 interviewed individuals, 1,220 (82,7%) recognized the scenario as a stroke. There were 3 words to correctly identify (based on correct intended response and spontaneously evoked associated symptoms) the stroke, which were “AVC” (acronym for cerebrovascular accident, in Portuguese), “derrame” (spillage) and “trombose” (thrombosis). There were significant differences among them concerning demographic, economic, educational and geographical aspects, but there was no difference according to the intended reaction among them. The most cited associated symptoms (excluding those present in the case) were impaired consciousness (10.6%), headache (8.9%) and dysesthesia (7.7%). “Aneurisma” (aneurism) was also cited, by 3 individuals.

**Conclusion:**

There are at least three words for stroke in Portuguese (“AVC”, “derrame” and “trombose”); they were similar in terms of correct intended responses and spontaneously cited accompanying symptoms. Stroke campaigns should apply different names to reach a broader audience and to improve stroke recognition.

## Introduction

The first step in the stroke chain of survival is recognizing the stroke symptoms and subsequently transporting the patient to a stroke center. This prehospital phase is the longest in the first hours of stroke [1]. Populational knowledge campaigns [2] and training the emergency medical service (EMS) members [3] may improve stroke recognition and minimize the time between symptoms onset and both stroke center arrival and reperfusion therapy delivery.

A chief cause for non-recognizing a stroke is atypical presentation, mainly posterior circulation syndromes and language deficits [4]. Studies have shown that the rate of undiagnosed strokes may dimmish whether one is applying wider algorithms, such as the BE-FAST (balance, eye, face, arm, speech, time) more than the FAST. This can be applied not only to health professionals but to the general population.

Some sociodemographic characteristics, like more educational years, better economic status, and previous experiences may influence the patient’s and bystander’s ability to recognize [5] and properly respond to a stroke [6]. However, other factors may play roles in this process, such as communication issues among patient/bystander and EMS dispatchers. Previous studies with the Spanish-speaking population showed at least 8 words to name a stroke [7]; in Portuguese, there were up to 28 [8] names for it. But it is not clear if these different words are related to the idea of the stroke itself or to other medical conditions, such as myocardial infarction, intoxications, or a malaise [9]. The impact of this kind of misconception on help-seeking behavior is likewise unknown. On the other hand, identifying the clinical significance of those terms and applying them in stroke awareness campaigns on lay media could maximize knowledge acquisition and reach broader audiences.

We conducted an exploratory study in the Northeast of Brazil, which presented a typical stroke clinical case to volunteers. We aimed to identify the different words used to name a stroke in Brazil, applying an open-ended survey; we assessed the concept of stroke through both the cited accompanying symptoms and intended reactions to this hypothetical scenario.

## Methods

This was a cross-sectional study, which data were collected from October 14th to November 30th, 2020. Our collection tool was developed by us, based on previous studies [10, 11]. It consisted of a typical clinical case of stroke that was read to the participants (You are in a conversation with your aunt and suddenly she presents difficulty speaking and walking. While you try to help her, you notice that her face is drooping, there is some weakness in her right arm and leg and you cannot understand what she tries to say), followed by 9 open-ended questions about it, besides the participant’s demographical data. From those questions, 3 are presented here: (a) “what do you think is happening to your aunt?”, (b) “in this situation what would you do?” and (c) “which other symptoms could happen in this condition?”.

Interviewers were trained medical undergraduate students. They approached passers-by in high-flow public spaces, such as squares, bus/subway stations, and street markets (similar to previous studies [8, 12, 13]), of pre-determined cities and invited those individuals to enroll in the study, configuring a quasi-random sample. The answers were completely spontaneous without any suggestion or interference from the interviewers. Sample size was not calculated prior to data collection. The responses were tape-recorded and transcribed to a digital registry. The volunteer could provide more than one answer to each question.

Inclusion criteria were being 18 years old or more; exclusion criteria were being tourists, having communication problems that impaired the interview or a personal history of stroke.

All Brazilian ethical standards were followed, and all participants gave their consent before the study (approval number: 4.299.487).

### Recognition and reaction to stroke

After being exposed to the clinical case, individuals were asked “what do you think is happening?”. All answers were registered, but those we considered correct during the study design were “AVC” or “acidente vascular cerebral/encefálico” (cerebro/encephalic-vascular accident), “derrame” (spillage), and “aneurisma” (aneurism). “Trombose” (thrombosis) was not expected by us in the study design phase, but was added as a correct answer once it met our criteria of proper both intended response and cited accompanying symptoms.

Then, we questioned “what would you do in this situation?”. Correct responses were calling an ambulance (SAMU, the acronym for Mobile Emergency Care Service in Portuguese, equivalent to EMS) or taking the patient to a hospital. Other answers were considered incorrect.

### Associated symptoms

The last question we examined was “Beside those symptoms presented (dropping face, weakness in arms and legs, troubled speech), could you cite other symptoms related to this condition?”.

The answers were grouped by the main author (MLM, blinded to the recognition and reaction answers) in 8 axes, as follows: impaired consciousness or cognition, headache, dysesthesia or paresthesia, cardiac-related, dizziness or incoordination or imbalance, visual problems, seizures and dysautonomia.

Answers such as somnolence, confusion, disorientation, fainting, and memory issues were labeled as impaired consciousness or cognition. We considered cardiac-related answers like tachycardia, dyspnea/breathlessness, chest pain, and high blood pressure. Visual problems were considered diplopia, blindness and impaired seeing. Sweating, chilling, vomiting, paleness and sphincter relaxation were labeled as dysautonomia. The other axes are self-explained.

### Statistical analysis

Data were analyzed to assess the concepts associated with the different words used to describe the stroke. This was done through an analysis of the intended reaction (question b) and of the symptoms spontaneously evoked (question c).

The four most frequently cited responses (AVC, derrame, infarto and trombose) were analyzed according to sociodemographics (age, formal school years, sex, race, having private health insurance), geographic site of data collection (state’s capital, and Bahia state, the state with the highest number of respondents), intended reaction to the stroke scenario and each of the 8 axes of evoked related symptoms.

For qualitative variables, data are presented as absolute numbers and percentage, and Fisher test was applied; size effect is presented as relative risk and 95% confidence interval. For quantitative variables, data are presented as median and interquartile range (IQR), and the Mann-Whitney test was applied. All variables were handled as dichotomic, except age and school years.

We provide the results of all correct responses (as a whole) to act as a reference, and each specific name was compared to all other correct names. Data from those who did not recognize the clinical case as stroke are not presented here (except for the “infarto” group).

Data were assessed in the IBM Statistical Package for the Social Sciences version 25 for MacOS. Missing data were < 0.1% and were excluded from the specific analysis. The significance level of all statistical tests was set at p < .050 (two-tailed).

## Results

The volunteers were recruited in 12 cities of Brazil’s Northeast (median 657,000 [IQR = 104,000-958,000] inhabitants per city), including 6 (Maceió, Natal, Recife, Salvador, São Luiz, Teresina) out of the 9 states’ capitals. Table 1 shows the demographic and geographic characteristics of our sample.

**Table 1:** Sample characteristics and comparison with Brazil’s Northeast data

Variable		n = 1,220	Northeast (%)*
Age**		32 (24–45)	-
	18–39^#^	926 (75.9)	34.3
	40+	549 (24.1)	39.4
School years**		12 (11–16)	-
	0–11^#^	585 (39.7)	60.7
	12+	890 (60.3)	39.7
Sex, female^#^		660 (54.1)	51.9
Race, White^#^		397 (32.5)	24.7
Have private health insurance^#^		428 (35.1)	-
From the capital of a state^#^		747 (61.2)	-
From Bahia state^#^		248 (20.3)	-

*census data from Instituto Brasileiro de Geografia e Estatistica (IBGE), 2019 (available at https://sidra.ibge.gov.br/)

**median (IQR), ^#^n (%), - = not available data

**Fig. 1:**
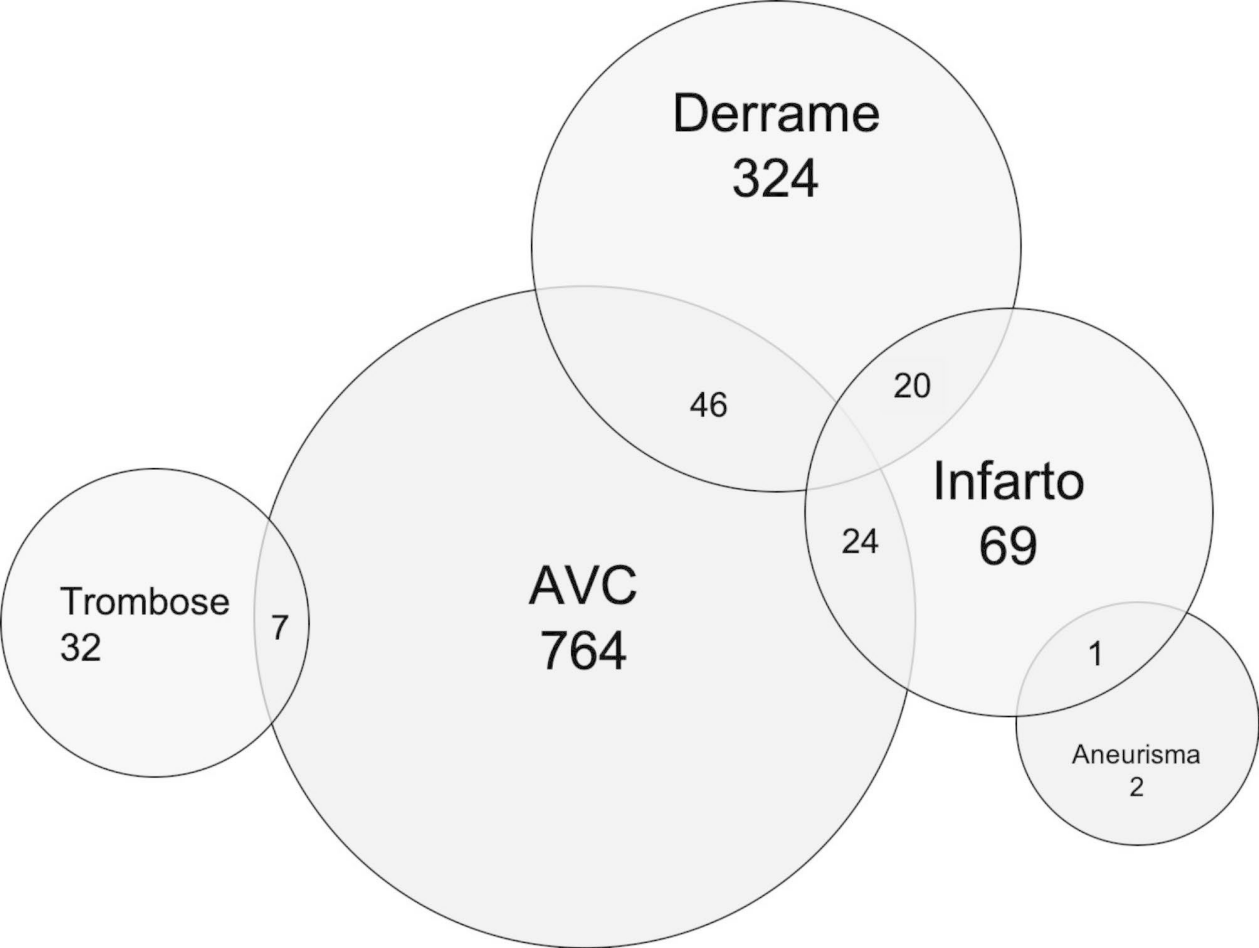
Venn’s diagram showing the relations among the answers to the question “what do you think is happening?”. Some volunteers answered more than one name to our inquiry

Various responses were given to the question “what do you think is happening?”. The answers considered correct were “AVC” or “acidente vascular cerebral” (n = 823), “AVE” (n = 21), “derrame” (n = 390), “trombose” (n = 39) and “aneurisma” (n = 3), totaling 1,220/1,475 (82,7%). Other responses were “infarto” (n = 114, infarction), “paralisia” (n = 13, paralysis), “ataque cardíaco” (n = 6, heart attack), “convulsão” (n = 6, seizures), and in lower frequency tumor or cancer, “fraqueza” (weakness), Parkinson’s or Alzheimer’s disease, “passamento” (malaise), “depressão” (depression), “esclerose” (sclerosis), and “some disease/sickness”. In Fig. 1 we show the relations among some answers to this inquiry.

In Table 2 we show the substantial sociodemographic and regional/geographic differences among the three most cited names for stroke (“AVC”, “derrame” and “trombose”) and “infarto”.

**Table 2:** Bivariate analysis of sociodemographic, geographic and intended response, according to the words used to identify the clinical presentation of stroke

Variable	AVC (n = 841)	Derrame (n = 390)	Infarto (n = 114)	Trombose (n = 39)
Age, median (IQR)	31 (23–44)***	33 (24–44)	35 (26–49)*	38 (30–55)*
School years, median (IQR)	12 (11–16)***	12 (9–14)***	12 (11–16)	12 (9–16)
Sex, female^#^	491 (58.4)***	179 (45.9)***	62 (54.4)	16 (41.0)
Race, White^#^	285 (33.9)	108 (27.7)*	30 (26.3)	14 (35.9)
Health insurance^#^	332 (39.5)***	104 (26.7)***	31 (27.2)	8 (20.5)*
Capital of a state^#^	546 (64.9)***	218 (55.9)*	65 (57.0)	11 (28.2)***
Bahia state^#^	141 (16.8)***	115 (29.5)***	35 (30.7)*	0 (0.0)***
Reaction, call EMS^#^	378 (44.9)	156 (40.0)	48 (42.1)	14 (35.9)
Reaction, take directly to a hospital^#^	328 (39.0)	167 (42.8)	44 (38.6)	16 (41.0)

^#^n (%)

*p < .05, ***p < .001, indicates analysis of the specific column vs. all other answers

From those individuals who identified properly the stroke (given any correct name to it), a higher proportion opted for calling EMS (530, 43.4%) than taking the patient directly to a hospital (487, 39.9%, RR = 1.10, 95%CI = 1.05–1.16, p < .001). However, the intended responses to the stroke were similar in each of the name groups (Table 2).

Likewise, in Table 3 we depict the symptoms that volunteers spontaneously cited as being related to a stroke. The most frequently reported symptom – impaired consciousness – was mentioned by only 129/1,220 (10.6%). “AVC”, “derrame” and “trombose” had a similar profile of accompanying symptoms, such as headache, paresthesia and impaired consciousness. However, the word “infarto” was significantly more associated with cardiac symptoms, in tandem with few reports of headache and impaired consciousness, which lead us to think that “infarto” is not a word for stroke.

**Table 3:** Bivariate analysis of spontaneously-evoked related symptoms, according to the words used to identify the typical stroke clinical case

Other symptoms	Correct answer (n = 1,220)	AVC (n = 841)	Derrame (n = 390)	Infarto (n = 114)	Trombose (n = 39)
Impaired consciousness or cognition	129 (10.6)	91 (10.8)	36 (9.2)	6 (5.3)*	3 (7.7)
Headache	108 (8.9)	85 (10.1)*	24 (6.2)*	5 (4.4)*	1 (2.6)
Dysesthesia or paresthesia	94 (7.7)	75 (8.9)*	19 (4.9)*	14 (12.3)	2 (5.1)
Cardiac-related	79 (6.5)	49 (5.8)	27 (6.9)	30 (26.3)***	3 (7.7)
Dizziness or incoordination or imbalance	64 (5.2)	49 (5.8)	14 (3.6)	8 (7.0)	2 (5.1)
Visual problems	33 (2.7)	23 (2.7)	11 (2.8)	2 (1.8)	0 (0.0)
Seizures	29 (2.4)	22 (2.6)	9 (2.3)	4 (3.5)	1 (2.6)
Dysautonomia	29 (2.4)	22 (2.6)	7 (1.8)	5 (4.4)	1 (2.6)

Other symptoms mean symptoms not presented in the presented clinical case

Correct answer column describes characteristics of all correct answers (AVC, derrame, trombose and aneurisma) as a whole

*p < .05, ***p < .001, indicates analysis of the specific column vs. all other correct answers, as a reference group

“AVC” group was younger, had higher years of education, economic status (inferred by private health insurance) and frequency of women; those individuals more often lived in capitals.

“Derrame” was quoted often in Bahia and in non-capitals. This group was made mostly of non-Whites, men, with lower levels of education and economic status. The accompanying symptoms cited were in general similar in the “AVC” and “derrame” groups.

“Trombose” was cited mainly in the states of Paraíba (n = 17), Ceará (n = 11), but also Maranhão, Piauí and Rio Grande do Norte in lesser frequency. Other states did not mention this name. Individuals in this group were significantly older, lived away from capitals and had no health insurance. There was no difference concerning related symptoms.

The group that responded “infarto” had a higher chance of stating that cardiac symptoms were related to the case (RR = 1.35, 95%CI = 1.18–1.54, p < .001), in tandem with significantly less headache and impaired consciousness. They were older and lived in Bahia more often.

## Discussion

As far as we know, this is the largest Brazilian study assessing the knowledge of the general population about stroke, and the first exploring the different words to name a stroke. Considering that there was no difference in the intended reaction and a similar profile of satisfactory spontaneously-evoked related symptoms, we consider that “AVC”, “derrame” and “trombose” are equivalent words used to describe a stroke in this population. It is worth noting that there are significant influences of geographic/regional, sociodemographic and probably cultural differences in the use of these names, then populational campaigns about stroke should apply them in order to reach a wider audience. Another word to describe the situation we presented, “infarto”, seems to be a misconception of the clinical case and is related to myocardial infarction. The awareness of other stroke warning signs was found to be inadequate, emphasizing the necessity for additional studies and targeted campaigns to address this issue..

There are 17 MeSH terms for stroke in Portuguese [14], but they could be summarized in 3 sets: “AVC”, “ictus” and “apoplexia”. “Trombose” is not listed and “derrame” is only listed under the heading “hemorrhagic stroke”. The first word in the History of Medicine describing a stroke, apoplexy [9], is not anymore used in this sense – it was a generic term referring to a sort of diseases which have in common a sudden onset (such as pulmonary embolism and myocardial infarction). A review among Spanish-speaking populations found that there were at least 8 words to stroke [7], including “trombosis”, “infarto cerebral”, “derrame cerebral” and “embolia”. Those observations highlight the notion that languages are alive and evolving (considering the Latin origin of Portuguese and Spanish, likewise similarities with English words), and that pathophysiological mechanisms – like embolism, aneurysm, thrombosis, and spilling (“derrame”) of blood – may influence disease naming.

Some Brazilian studies recognized “derrame” [8, 10, 12, 13, 15] as a common alternative name for stroke. Indeed it is widely used in lay media [14]. This was cited mostly by non-White, less-educated men who also lived away from capitals, and without health insurance. Also, “derrame” was often used in Bahia state.

Nevertheless, only one study identified “trombose” [8], cited by 9.9% of their respondents; of note, that study also collected data in the Northeast, notably in the Ceará state. We collected data in three cities of Bahia state, but no one named “trombose” there. We also found particular geographical and socio-economical characteristics in this group.

Those educational, economical, racial, geographical and probably cultural differences among the names identified point out various aspects shaping the populational experience and the words for stroke. We highlight that these informal words for stroke in Brazil (“derrame” and “trombose”) were cited mostly by “minorities” (Blacks, poor ones, and undereducated), who are at higher risk for stroke [16] and might not be reached in campaigns that do not apply those terms. Thus, campaign developers should be aware of these alternative terms and use them, in order to maximize the target population and guide enhancing previous knowledge more than providing new information. Besides, EMS workers and physicians should keep in mind the possibility of stroke even in calls for other reasons. It is possible that there are other words for stroke that we did not identify.

Interestingly, the study of Pontes-Neto et al. [8] considered that “infarto” was a synonym for stroke, whereas other authors [10] and us believe that “infarto” is a misinterpretation of myocardial infarction and not a stroke recognition. Our approach of spontaneously-evoking related symptoms revealed more cardiac, besides less stroke-associated symptoms in the group that described the clinical scenario as “infarto”. However, as shown in Fig. 1, some individuals answered “infarto” and “AVC”, “derrame” or “aneurisma”, indicating a confusion about these disorders amongst the general population. Of note, one Brazilian study found that 30% of volunteers stated the heart was the organ affected in a stroke [17]. Furthermore, we found no difference in the intended reaction concerning all these words, indicating a notion of similar disease severity.

Knowledge about other symptoms related to stroke was poor, however similar to another study that applied a strategy similar [10] to ours (the main cited symptoms were headache and cognition problems, both by 8.1% of their sample). In general, the most common signs and symptoms cited in Brazilian studies applying an open-ended approach [12, 13, 17, 18] are motor (facial drop and/or hemiplegia), language impairment, headache and paresthesia, with a frequency around 30 − 20%. This frequency was similar in developed countries [7, 19, 20]. Recall of symptoms related to posterior circulation is even poorer.

In Brazil, the SAMU acronym is the equivalent of the FAST (face, arm, speech, time) algorithm, which has been applied in stroke educational campaigns, focusing on those symptoms. Recently, considering that symptoms of posterior circulation were underrepresented in those campaigns, the acronym evolved to BE-FAST (adding balance and vision-related symptoms), which improved stroke accuracy by 10% [21]. To the best of our knowledge, this addition was not yet applied in Portuguese-speaking populations for campaign purposes. Considering that balance and eye alterations were cited by only 5% and 3% of our sample, respectively, it is important to add these symptoms to stroke campaigns and train emergency physicians to enhance stroke recognition scenarios. Furthermore, how to recognize and react to a stroke should be integrated into the school curriculum [22]. However, recognition of stroke warning signs might not be related to early arrival in stroke centers [23].

Future research should investigate the impact of adding “alternative”, non-technical, words in stroke campaigns regarding the outcomes of patients, including in other countries. Likewise, implement regional versions of BE-FAST and assess the levels of retention of this more complex screening tool.

Our study has a number of limitations. Firstly, our sample is young and highly educated, which may not represent the Brazilian population. Second, we cannot rule out that there are other words that accurately describe a stroke in Brazil. “Trombose” and “aneurisma” were underrepresented in our sample and it is possible that small differences in these name groups would be found in larger samples, concerning sociodemographic, geographic, and clinical aspects.

## Conclusion

There are at least three words for stroke in Portuguese (“AVC”, “derrame” and “trombose”); they have a similar profile of spontaneously-evoked associated symptoms and correct intended responses. However, knowledge about associated symptoms (especially those related to posterior circulation) was poor and this issue should be addressed in populational campaigns. Physicians and EMS workers should keep in mind that stroke may be referred to as other names. Other countries should look for other words that lay population use to describe a stroke.

Our data highlight the need for assessing the benefits of the BE-FAST algorithm in our population.

## Data Availability

Data collected for the study, which were presented in this publication, including de-identified individual participant data can be made available to other researchers from the corresponding author on reasonable request and after signing appropriate data sharing agreements and providing approval from respective ethics boards. A data dictionary defining each field in the set also can be provided.
